# Does Sexual Desire Fluctuate More Among Women than Men?

**DOI:** 10.1007/s10508-022-02525-y

**Published:** 2023-01-25

**Authors:** Emily A. Harris, Matthew J. Hornsey, Wilhelm Hofmann, Patrick Jern, Sean C. Murphy, Fanny Hedenborg, Fiona K. Barlow

**Affiliations:** 1 Melbourne School of Psychological Sciences, University of Melbourne, Parkville, 3010 Australia; 2 Business School, University of Queensland, Brisbane, QLD Australia; 3 Faculty of Psychology, Ruhr University Bochum, Bochum, Germany; 4 Department of Psychology, Åbo Akademi University, Åbo, Finland; 5 School of Psychology, University of Queensland, St Lucia, QLD Australia

**Keywords:** Desire, Sex differences, Gender differences, Experience sampling, Panel data, Relationship science

## Abstract

There is a lay assumption that women’s sexual desire varies substantially over time, whereas men’s is stable. This assumption is mirrored in prominent theories of desire, which posit that women are more variable than men in the extent to which they desire sex, and that women’s sexual desire is more contextually sensitive than men’s. We tested this assumption across three longitudinal studies. Study 1 assessed desire at 3 time points spanning 13 years (*N*_observations_ = 5562), and Studies 2 and 3 (*N*_observations_ = 11,282) assessed desire moment-to-moment over 7 days. When desire was measured over years, women were more variable in their sexual desire than men (Study 1). However, we found a different pattern of results when desire was measured over the short term. In Studies 2 and 3, we found no significant differences in women’s and men’s desire variability. The extent to which desire varied as a function of affective states (e.g., happiness) and relationship-oriented states (e.g., partner closeness) was similar for women and men, with some exceptions; women’s desire was more negatively associated with tiredness and anger in Study 2. These data qualify existing assumptions about sex differences in sexual desire variability.

## Introduction

There is a lay assumption that men experience stable and high sexual desire, an attribute inherent to “maleness” (Regan & Berscheid, 1995). Women’s sexual desire, on the other hand, is thought to be more variable because it is more readily influenced by psychological and situational factors. For example, it is assumed that women’s desire may change from moment to moment depending on factors such as how desirable they feel, or how they feel about their relationship (Regan & Berscheid, 1995).

The assumption that women have a weaker and more sensitive sex drive than men has been echoed in social, evolutionary, clinical, neuro-, and relationships psychology (e.g., Birnbaum et al., 2016; Diamond, 2003; Everaerd et al., 2006; Gonzaga et al., 2006). Reflecting on his discussions with men and women about sexual desire, the psychiatrist Levine (2003) concludes that “male drive, motivation, and wish for sex lasts longer in the life cycle and is far more reliably present and intense. Female sex drive, being weaker, is more easily ignored by women and eradicated by social circumstances” (p. 281).

The assumption that women’s desire is more sensitive to social context, and hence more variable, seems to apply to both long- and short-term instances of variability. Women’s desire is thought to be more sensitive to major life events and changes in cultural context, which is reflective of longer-term variability measured over several years (Baumeister, 2000). Women’s desire is also theorized to change more in response to immediate circumstances. Previous theorizing suggests that “if women are malleable in response to situational and social factors, then as a woman moves from one situation to another, her sexual desires and behaviors may be subject to change…male sexual patterns will remain more stable and constant across time and across different situations” (Baumeister, 2000, p. 348). The variability described here suggests short-term change, that is best captured by a fine-grained assessment of desire as it happens “in the moment” over several days.

However, there is limited empirical evidence supporting the assumption that women’s desire fluctuates more than men’s desire. While there are notable studies of desire over time (e.g., Impett et al., 2008; Mark, 2012, 2014; Muise et al., 2016; Raisanen et al., 2018), few studies directly address gender differences in desire variability. Does women’s desire vary more than men’s over the lifespan? And does it vary more across the short term, from situation to situation?

Our primary aim is to test whether there is greater variability in women’s sexual desire compared to men’s over the long and short term. Relatedly, we test the idea that women’s desire is more contextually sensitive compared to men’s (i.e., is women’s desire more likely to fluctuate in response to other momentary states?). Below, we discuss theoretical arguments and empirical evidence for differences in women’s and men’s desire variability. Before starting, however, it is important to discuss what we mean when we refer to women and men, desire, and desire variability.

### Gender and Sex

Above, we quoted from theories that refer to both gender (women and men) and sex (females and males) differences in desire variability. It is common in the literature and everyday discourse to conflate gender and sex, and while they are related, they are not the same (e.g., see van Anders, 2015). A person’s gender may refer to their identity as a woman, man, non-binary, or allo-binary person. This identity may incorporate social and cultural meanings of gender. A person’s sex tends to refer to identities that are based on biology, including hormones, genitalia, and genes (van Anders, 2015). In the present studies, we measure sex. Thus, when referring to the present studies, we will refer to sex differences. We will use the terms “women” and “men” for readability; however, it is important to note that our samples may include females and males who do not identify as women and men. When referring to previous literature, we will refer to gender or sex differences in desire, depending on which has been studied.

### Sexual Desire

Sexual desire refers broadly to an interest in some form of sexual activity (Mark & Lasslo, 2018; van Anders et al., 2021). Desire may be experienced as an interest in being sexual with other people or solo sexuality (Spector et al., 1996) and may reflect an interest in different aspects of sex, including in-person sex, media-based sex, and/or sexual fantasy (Chadwick et al., 2017; Gormezano et al., 2022; van Anders, 2015). The multifaceted nature of desire is reflected in numerous measures of desire, which can measure desire for a partner (Apt & Hurlbert, 1992), desire for solo sexuality (Spector et al., 1996), or desire in response to viewing erotic stimuli (Dawson et al., 2013; Goldey & van Anders, 2012).

Some studies assess “trait-like” desire, whereby desire is measured at a single time point and is assumed to be indicative of a person’s general level of desire. Other studies assess state desire, typically over multiple time points, assuming that desire can change from moment to moment. Here, we assess state desire to test whether women’s desire is more likely to vary (i.e., is more state-like) than men’s desire.

### Desire Variability

Broadly, desire variability refers to the extent to which a person’s desire changes over time. One common measure of variability is the standard deviation (SD), which is the average extent to which a person’s scores on desire deviate from their mean score (Mestdagh et al., 2018). The standard deviation is referred to as *net variability* (Koval et al., 2021). A second, related measure is the mean squared successive difference score (MSSD). In contrast to the standard deviation, the MSSD takes into account the temporal order of change—it is the average magnitude of change from one time point to the next, and is referred to as a measure of instability (Koval et al., 2021).

**Fig. 1:**
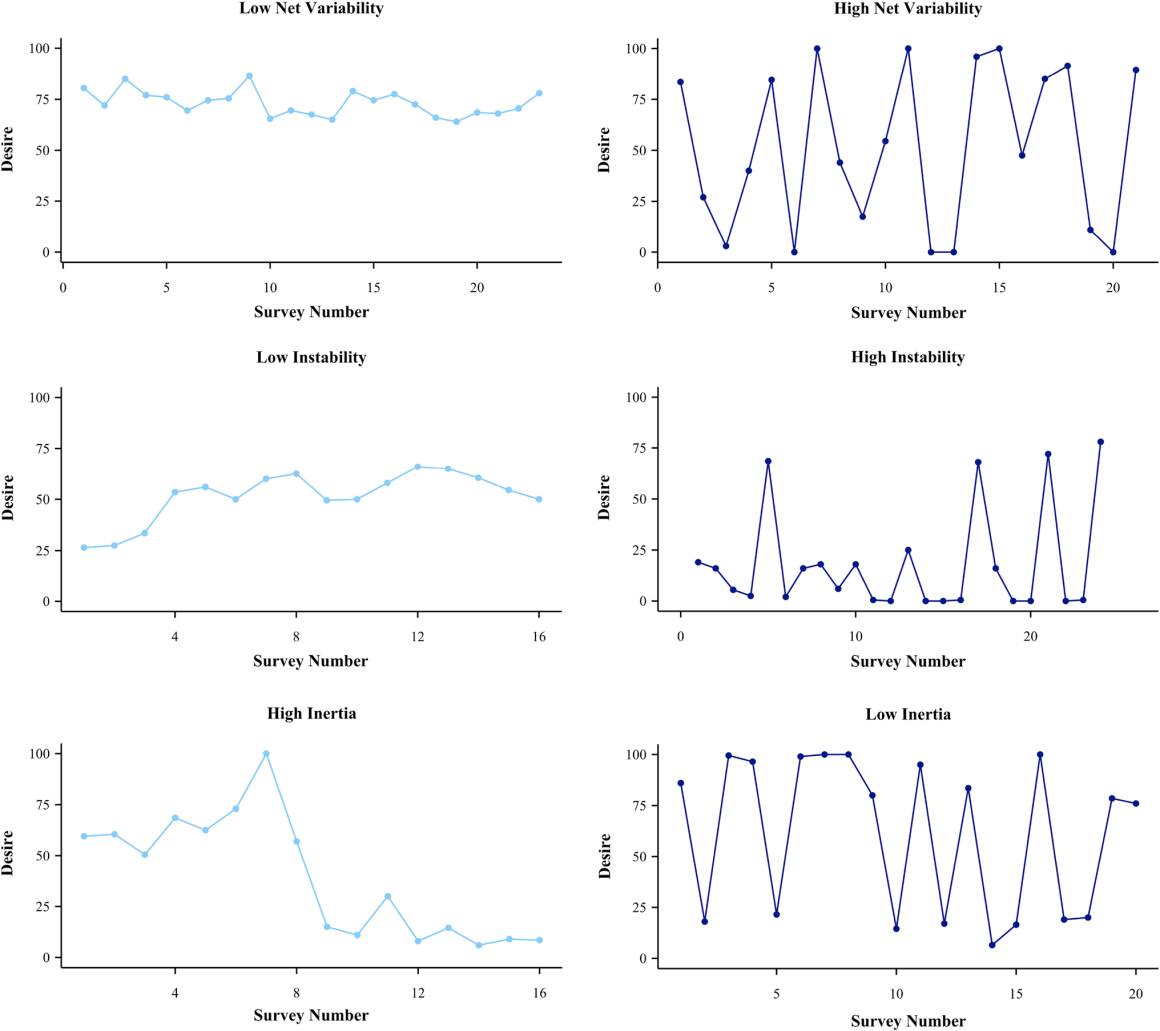
Time series plots of participants with high and low desire inertia, net variability, and instability in Study 3. *Note*: Net variability is calculated using relative standard deviations (relative SD), instability is calculated using the relative mean squared successive difference score (relative MSSD), and inertia is calculated using the autoregressive (AR1) slope. Inertia refers to persistence over time

A third, more recent measure of variability is inertia. In contrast to net variability and instability, inertia is a measure of whether something is resistant to change. For example, if a person’s desire at one time point tends to be similar to their desire at the previous time point, there is a high degree of inertia. Net variability, instability, and inertia are overlapping measures of variability that broadly assess the degree of change in an outcome (Koval et al., 2021). In this study, we will assess all three estimates of variability: net variability, instability, and inertia, to provide a nuanced picture of how desire may change over time for women and men (Dejonckheere et al., 2019; Koval et al., 2021). We present plots of participants with high and low scores on desire inertia, net variability, and instability, in Fig. 1.

Having defined our key terms, we will now turn to theories of gender and sex differences in desire variability. Our review focuses largely on desire among heterosexual, cisgender women and men since they have received the most attention in previous research though with some exceptions (e.g., Chadwick et al., 2017; Jodouin et al., 2021; Mark et al., 2018).

### Gender and Sex Differences in Desire Variability

One of the most prominent theories of within-person changes in sexuality over time is erotic plasticity theory (Baumeister, 2000). According to erotic plasticity theory, women are more variable than men on three dimensions: sexual attitudes, sexual behavior, and sexual desire. In developing the theory, Baumeister (2000) reviewed research on changes in women’s and men’s sexual expression over time, focusing on intra-individual change. The review found that over time, women were more likely to exhibit substantial changes in sexual attitudes than men (e.g., in terms of how sexually permissive they are) and were more likely than men to show variation in sexual behavior (e.g., going from regular sexual activity to a sexual “drought,” or exhibiting fluidity in sexual orientation/identity). The greater variability in sexual attitudes and behaviors among women was theorized to extrapolate to desire, such that “the average man’s desires should remain more stable and constant than the average woman’s” (Baumeister, 2000, p. 348).

Baumeister (2000) presented three possible reasons for why women show greater erotic plasticity compared to men. First, men have a higher sex drive compared to women, and due to evolutionary pressures, men’s sex drive may be driven more strongly by biological than social factors. Second, because men have greater physical and sociopolitical power relative to women, men are able to coerce or force women to have sex in ways that men desire. As such, greater sexual malleability in women may be an adaptive response to men’s greater power. Finally, erotic plasticity may be a function of women’s need to switch from “no” to “yes” when considering potential partners. Due to the different reproductive costs for women and men, men have evolved to be open to sexual opportunities, whereas women play the role of the sexual gatekeepers, who are choosy about when and with whom they are interested in sex.

However, the theorizing around erotic plasticity was constrained by existing evidence. At the time, the evidence was heavily weighted on the first two dimensions of sexuality: attitudes and behavior. In contrast, there was no quantitative research on within-person changes in sexual desire. In his review, Baumeister (2000) notes: “the measurement of sexual desire … is undoubtedly more difficult than the measurement of behavior or attitudes, and so it has received less study… Given the present state of the evidence, the gender difference in erotic plasticity is far better supported with respect to attitudes and behavior than desire itself” (p. 364). Below, we review the work that has since been done on gender and sex differences in desire variability. We review work on medium- to long-term changes in desire, over the course of several years, and short-term changes from moment to moment.

### Medium- to Long-Term Desire Variability

Sex and relationship researchers have explored how and why desire might change over the lifespan and during life phases such as parenthood. McNulty et al. (2019) directly addressed the question of differences in women’s and men’s desire variability by examining changes in sexual desire in newlywed couples. Across two studies spanning approximately 4 and 4.5 years, women’s desire declined over time, whereas men’s desire remained relatively stable. This sex difference in desire variability was partly explained by childbirth, whereby women who gave birth showed steeper declines in desire, and this effect was not present for men. This study provides the first quantitative support for the assumption that women’s sexual desire is more variable than men’s desire, at least in the medium to long term among newlywed heterosexual couples.

This gender difference in desire variability has been replicated in a study of new parents (Rosen et al., 2021; also reported in Rossi et al., 2022). Rosen et al. (2021) measured sexual desire among 203 couples from 20 weeks into pregnancy to 12 months postpartum. The majority of participants identified as heterosexual (90–95%), and results were consistent when including only heterosexual couples.^1^ Mothers’ desire decreased between mid-pregnancy to 3 months postpartum and increased from 3 to 12 months postpartum. Men partners’ desire was stable during this period.

Together, these studies suggest that mothers’ desire is more likely to change during the transition to parenthood compared to fathers’ (McNulty et al., 2019; Rosen et al., 2021). These findings are consistent with the gendered experiences of childbirth and parenthood. Women partnered with men experience the physical effects of pregnancy, childbirth, and the postpartum period and tend to take on the majority of childcare (Harris et al., 2022). Thus, it is difficult to assess whether there are gender differences in desire variability, independent of the gendered effects of parenthood (at least among women partnered with men).

To study desire over the lifespan, several studies have used cross-sectional data to compare people at different stages of their relationship (e.g., Klusmann, 2002) or their life (e.g., Beutel et al., 2008; Eplov et al., 2007). In a study of 1,865 German students in relationships, relationship duration was negatively associated with the desire to have sex among women but not men. Focusing on age, Beutel et al. (2008) found that desire was lower among older participants, and this effect was stronger for women, whereby differences in desire between younger and older women were greater than they were for younger and older men. These cross-sectional studies provide preliminary support for the theory that women’s desire is more variable than men’s desire over the course of years. However, because this data is cross-sectional, it cannot assess within-person change in desire and, as such, cannot address variability.

### Short-Term Desire Variability

There is a growing literature examining sexual desire over the short term, explicitly measuring desire as a “state” that can change from moment to moment (for review, see Mark & Lasslo, 2018). One early study by Ridley et al. (2006) measured “marital lust” among heterosexual couples across 56 days. To assess patterns of fluctuations in desire, they performed a cluster analysis based on the degree of variation in lust over time. The distribution of men and women between the clusters was almost identical: Nine men and nine women were categorized as having “stable” or “slightly fluctuating” lust, and 14 men and 13 women were categorized as having “moderate” and “highly fluctuating” lust. However, due to the small sample, statistical comparisons of the groups were not conducted.

In a more recent study, Vowels et al. (2018) analyzed patterns of change in desire over 30 days. Again, this study did not statistically test for gender differences in desire variability, but the authors commented on the similarities in women’s and men’s patterns of change. Women and men had similar average frequencies of change and showed similar levels of “persistence,” whereby women and men “peaked” in their desire at similar rates and maintained that level of desire for approximately 3 days.

Similarities between women and men have also been found when assessing desire change in response to viewing sexual stimuli. After viewing erotic stimuli, women and men report similar levels of desire (Both et al., 2004; Dawson & Chivers, 2014; Goldey & van Anders, 2012) and the effects of sexual stimuli on desire are similar for women and men (Goldey & van Anders, 2012). These studies assessed desire across two time points (pre- and post-stimulus), so do not assess desire variability per se, but do assess the extent to which desire may be responsive to stimuli. One exception is a study by Dawson et al. (2013) that assessed habituation in arousal. When repeatedly shown the same sexual stimuli, women and men showed a similar pattern of decline in their subjective arousal responses (Dawson et al., 2013). Subjective arousal is related to desire in that both can refer to sexual interest (Mitchell et al., 2014). These results suggest that short-term changes in arousal (and perhaps desire) in response to sexual stimuli may be similar for women and men.

We identified two studies that supported the theory that women’s desire is more variable than men’s desire over the short term. Diamond et al. (2017) examined daily variability in sexual attraction among 294 women and men. Women’s feelings of sexual attraction toward others were more variable than men’s feelings of attraction. Sexual attraction overlaps with sexual desire, whereby both refer to feelings of sexual interest in another person or other people. This study provides some indication that women’s attractions, and by extension, desire, may be more variable in response to situational factors that change over the course of days.

Second, Mark et al. (2019) assessed desire each day for 30 days among a sample of heterosexual couples. Men’s current desire was associated with their desire on the previous day, whereas women’s desire was not associated with their previous day’s desire. These findings suggest that men’s daily desire may be more stable relative to women’s; however, again, this study did not statistically compare the moderating effect of gender, and both effect sizes were small (0.01 for men and 0.04 for women). It is, therefore, unclear whether men’s desire was significantly more stable compared to women’s desire.

We also reviewed work looking at hormonal variability and sexual desire, since the menstrual cycle is one proposed cause of women’s desire variability (Vowels et al., 2018). However, the majority of the research on desire and hormones over time has included women only (e.g., Pillsworth et al., 2004; Röder et al., 2009; Roney & Simmons, 2013) or has not reported sex differences in desire variability, so cannot speak to the current research question. Other studies have examined changes in desire over the short term among women and men (e.g., Goss et al., 2022; Vowels & Mark, 2020; Kim et al., 2021; for reviews, see Dawson & Chivers, 2014; Mark & Lasslo, 2018), and while these studies typically report whether there are differences in average desire, they do not report whether desire variability is different for women and men. Rather, they tend to focus on predictors of desire, and we review this literature in the following sections on desire and mood and the relationship context.

In sum, there is some evidence that women’s desire is more variable over the medium- to long-term (McNulty et al., 2019; Rosen, et al., 2021). However, these findings are based on data from heterosexual newlywed couples and parents during the transition to parenthood. It is unclear whether these findings may generalize beyond these life phases. In our review of the literature on short-term desire variability, some studies suggested that patterns of change were similar for women and men (e.g., Ridley et al., 2006; Vowels et al., 2018), whereas, two studies suggested that men's desire was more stable than women's desire (Diamond et al., 2017; Mark et al., 2019). However, these studies did not statistically compare women and men’s desire variability. There is some evidence for a gender difference in the variability of sexual attraction (Diamond et al., 2017), but not arousal (Dawson et al., 2013), yet these studies did not assess desire directly. To our knowledge, there has been no systematic test of whether women’s desire is more variable than men’s desire when assessed throughout the day.

Below, we outline research addressing the separate and related question—also posed by erotic plasticity theory—of whether women’s sexual desire is more contextually sensitive than men’s desire. We first focus on the research linking sexual desire to affective states, which is somewhat limited, and then focus on literature assessing the link between sexual desire and feelings of relational intimacy and affection.

### Desire and Mood

As highlighted earlier, women’s sexual desire is assumed to be highly variable because it is especially responsive to social context (Baumeister, 2000). There is some support for the idea that general affective states have a different impact on desire for women and men. For example, anger has a stronger negative effect on women’s desire relative to men’s in the context of listening to an erotic audiotape (Beck & Bozman, 1995). Women are also more likely to say that they did not engage in sex for reasons relating to their mood (e.g., “I was not in the mood”) compared to their men partners, who were more likely to cite partner-based reasons (e.g., “My partner was too tired”; Mark et al., 2020). The negative effects of tiredness and loneliness on sexual desire have been discussed in clinical and theoretical work (e.g., Basson et al., 2004; Levine, 2003; Regan & Berscheid, 1995), but many of these studies focus only on women, and, to our knowledge, there has been no quantitative test of whether men’s desire is similarly impacted by tiredness or loneliness.

### Desire and the Relationship Context

The links between sexual desire and indices of relationship functioning have perhaps received the most interest from other literatures. One prominent social-evolutionary theory addressing gender differences in sexual desire is Diamond’s (2003) biobehavioral model of love and desire. Diamond (2003) hypothesizes that, while love and desire are independent phenomena, women are more likely to restrict their experiences of desire to contexts in which they are in love. This is because women have been socialized to confine their desires to committed, and ideally loving, relationships. Thus, women’s desire is expected to vary in response to feelings of love and attachment to a greater extent than men’s desire.

In line with this view, a group of clinicians have proposed the new view of women’s sexual desire (Tiefer, 2001) which levels a critique against the *Diagnostic and Statistical Manual of Mental Disorders* (DSM) for developing a model of sexual dysfunction that does not account for relationship factors (among others), largely because the original model was man-centric. The implicit assumption is that relationship factors are more relevant when treating women’s sexual problems than they are for men’s sexual problems.

These perspectives converge on the idea that women’s sexual desire is more relational than men’s. However, evidence to support the idea that relationship factors—intimacy, closeness, security, and affection—are particularly strong predictors of women’s sexual desire is mixed. A number of studies have found that gender does not moderate the effect of relationship states on sexual desire (e.g., Birnbaum et al., 2016; Gonzaga et al., 2006; van Lankveld et al., 2018). For example, feeling motivated to meet a partner’s sexual needs and relationship intimacy are equally important predictors of men's and women’s desire (Muise et al., 2013a; Rubin & Campbell, 2012). In contrast, Impett et al. (2008) found that the positive association between approach relationship goals (i.e., striving for relationship growth) and daily sexual desire was larger for women than men.

Overall, while there is some evidence that women’s desire may be particularly sensitive to some relationship factors, the evidence provides only weak support for the theory that women’s desire is more contextually sensitive than men’s.

### The Current Study

In sum, our review of the theoretical and empirical literature on gender and sex differences in desire variability has led us to the following three research questions:

**RQ 1a**: Do women show greater variability in feelings of sexual desire over the long term compared to men?

**RQ 1b**: Do women show greater variability in feelings of sexual desire from moment to moment compared to men?

**RQ 2:** Compared to men, is women’s sexual desire more strongly related to their general affective states?

**RQ 3:** Is women’s sexual desire more strongly tied to relationship-oriented states than men’s?

While the theories of gender and sex differences in desire variability suggest that the answers to these questions are “yes,” we present them here as exploratory research questions, given the mixed empirical evidence for these theories. To address these research questions, we conducted three longitudinal studies measuring men’s and women’s desire. We tested our primary research question—does women’s desire vary more than men’s?—by assessing long-term variability in Study 1 (13 years) and short-term variability in Studies 2 and 3 (7 days). Studies 2 and 3 also measured concurrent affective states (e.g., happiness) and relationship-oriented states (e.g., relationship satisfaction) to assess whether women’s desire is more contextually sensitive than men’s (RQs 2 and 3). In Study 2, in addition to testing the effects of participants’ relationship-oriented states (e.g., their level of relationship satisfaction) on desire, we also examine partner effects (e.g., a partner’s level of relationship satisfaction) on desire. We conduct exploratory dyadic analyses to assess the extent to which desire may fluctuate as a function of a partner’s relationship and affective states.

## Study 1

Study 1 assessed desire variability across the long term using data collected across three waves of a Finnish population-based twin study spanning 13 years. We tested our first research question—does women’s sexual desire vary more than men’s?—across a significant cross-section of people’s adult lives. Previous theorizing suggests there will be gender differences in long-term variability in desire, with changes occurring as a function of moving country/culture, relationship dissolution, and sociopolitical changes, with larger effects expected among women. Additionally, there are social and biological changes specific to women, such as childbirth and menopause, that are captured with data spanning many years. Study 1 allows us to test for the effects of such changes. Study 1 does not address the second and third research questions regarding the effects of affective states and relationship factors on desire, since these measures were not included across waves.

### Method

#### Participants

The initial dataset comprised 13,829 twins. Participants were excluded if they did not respond to at least one of the three items measuring desire (*n* = 549), if their sex was not recorded (*n* = 8), and if they responded at only one or two of the three waves (*n* = 11,426). Participants were invited to take part in a raffle to win a gift card at each wave of data collection (e.g., in 2019, participants had the chance to win one of 100 × 25€ gift cards). Participants were assigned as either female or male by the Central Population Registry of Finland database. The sample included only people assigned female or male. We use the terms “women” and “men” for readability; however, the sample may include participants who are not cisgender. The final sample comprised 1,854 participants (*N*_men_ = 594, *N*_women_ = 1,260), and 5,562 observations. Wave 1 data were collected in 2006 for men (*M*_*age*_ = 27.14, SD_*age*_ = 4.56) and women (*M*_*age*_ = 26.29, SD_*age*_ = 4.97), wave 2 data were collected in spring 2012 for men and in fall 2013 for women, and wave 3 data were collected in 2019 for men and women.

#### Procedure

Data were collected as part of a larger study on genetic predictors of sexuality and aggression (for details, see Johansson et al., 2013). Participants were identified using the Central Population Registry of Finland and were invited to participate via mail. Participants who agreed were then provided with a link to the online surveys at each wave. Participants received a randomly generated eight-digit code in 2006 to ensure anonymity, and the same code was used in all subsequent data collections. Institutional ethics approvals for Studies 1–3 were received prior to data collection.

#### Measures

Measures included in Studies 1–3 are listed on OSF (https://osf.io/vsxwb/?view_only=fd32a4fe21d54631a30d7a93474154c9).

Partner desire was assessed in waves 1 and 2 using three items adapted from the Sexual Desire Inventory (Spector et al., 1996): “When you have sexual thoughts, how strong is your desire to engage in sexual behavior with a partner?”, “When you spend time with an attractive person (for example, at work or school), how strong is your sexual desire?”, and “Compared to other people of your age and sex, how would you rate your desire to behave sexually with a partner?” (2006: *α*_men_ = 0.74, *α*_women_ = 0.77; 2012/13: *α*_men_ = 0.77, *α*_women_ = 0.81; 2019). Response options ranged from 1 = No/low desire to 9 = Strong desire, and for the third item, 1 = Own desire much lower to 9 = Own desire much higher. In wave 3, the first item was not included in the survey, so a two-item scale was used (*r*_men_ = 0.48, *r*_women_ = 0.47). Results were unchanged when the two-item scale was used across waves 1 to 3.

#### Analytic Strategy

We assessed desire variability by calculating net variability and instability. Net variability is a person’s average deviation between each desire score and their mean level of desire (i.e., the standard deviation). We calculate the relative standard deviation because the absolute standard deviation is confounded by the mean (Mestdagh et al., 2018). For example, someone with a mean close to the upper or lower bound of a scale may have a lower absolute standard deviation than someone with a mean at the midpoint of the scale because their scores are less restricted by the scale bounds. Relative standard deviation accounts for differences in means by estimating the proportion of variance relative to the maximum possible amount of variance given a person’s mean score.

Second, we assessed instability, which refers to the average change between two successive time points. In contrast to net variability, which assesses net deviations from a person’s mean, instability takes into account the temporal order of change. Instability captures whether there are large swings in desire from one time point to the next. We calculated instability using the relative mean squared successive difference score (MSSD). Similar to relative standard deviations, each participant has a relative mean squared successive difference score, where higher scores represent greater instability.

The calculation of relative net variability and relative instability requires mean scores to be different from the lower and upper bounds of the scale (e.g., a participant cannot have a mean score on desire of 0 or 100). Participants whose mean desire was equal to the lower bound (*n* = 1) or upper bound of the scale (*n* = 9) were therefore excluded. To test for sex differences, we regressed sex on net variability and instability scores for desire. See Fig. 1 for examples of time series data from participants with high and low desire net variability and instability. We did not estimate desire inertia in Study 1 due to the small number of time points per participant.

Since twins were recruited for the study, we conducted multilevel models controlling for familial dependency by including a random intercept for family. We did not conduct twin-based analyses as heritability was not a focus of the study. In the following analyses, 0 = men and 1 = women. In Studies 1–3, we standardize all continuous variables and report standardized betas. Analysis code and output for Studies 1–3 are available on OSF (https://osf.io/vsxwb/?view_only=fd32a4fe21d54631a30d7a93474154c9).

In addition to our main tests for sex differences in desire variability, we conducted a series of sensitivity analyses. First, we tested the interaction between sex and number of children, since desire may be more variable among women as a function of having children (Rosen et al., 2021). We modeled the number of children participants had at Wave 3, and whether participants had children (dichotomous variable: 0 = no children, 1 = one or more children), as moderators of the associations between sex and desire variability.

Second, we tested for sex differences in variability for each item measuring desire separately. The two items included across all three waves of data collection measured conceptually distinct types of sexual desire: desire to have sex with a partner, and desire in the presence of an attractive person. Finally, since the sample included a larger proportion of women, we created a data set with a random sample of 600 women, such that we had approximately equal samples between sexes and re-ran our analyses.

### Results

Overall, mean desire across the 5,562 data points was close to the midpoint; *M* = 5.22, SD = 1.82. Men’s desire (*M* = 5.93, SD = 1.58) was significantly higher than women’s (*M* = 4.94, SD = 1.82), *β* = -0.55, SE = 0.04, *p* < 0.001, 95% CI = [ − 0.62, − 0.48].

#### Desire Variability

**Fig. 2:**
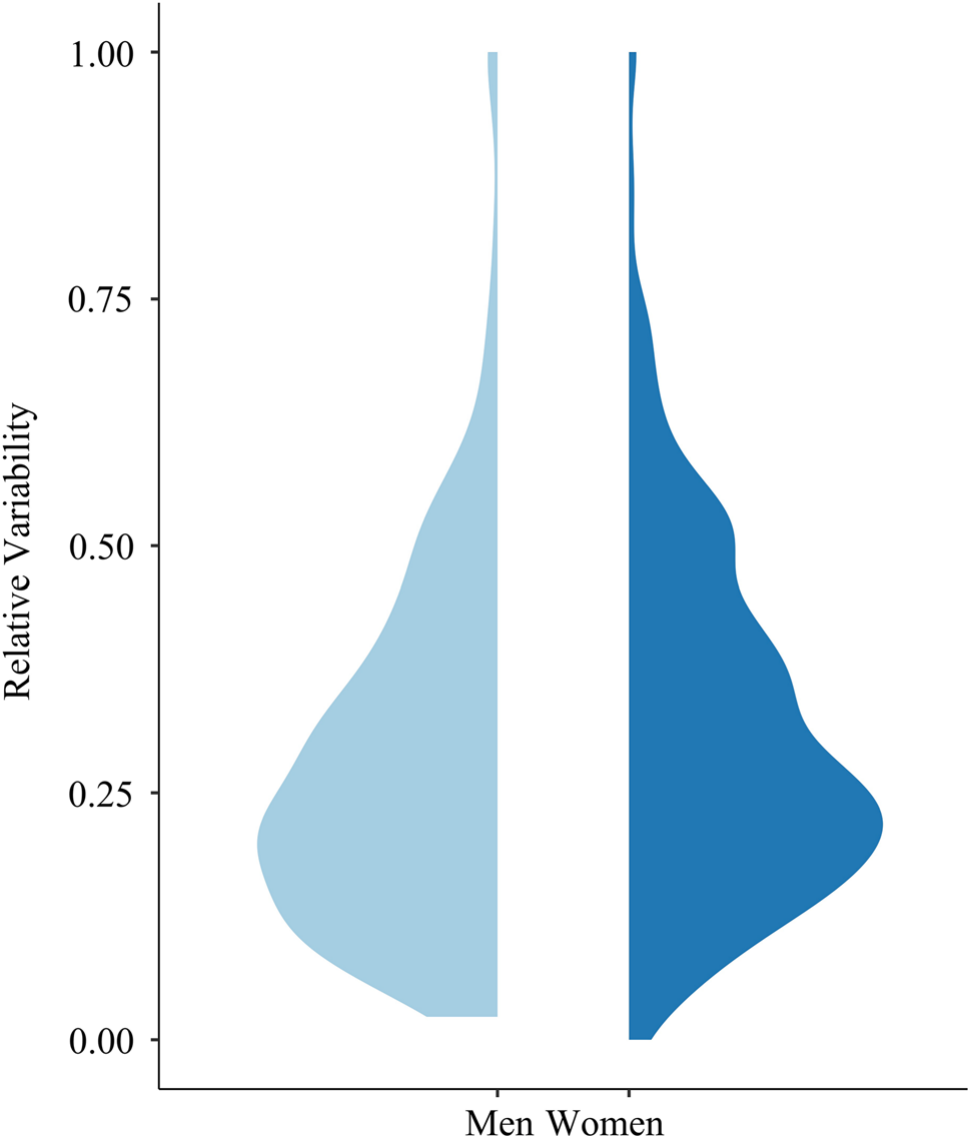
Distributions of net variability in sexual desire for men and women in study 1. *Note*: Net variability refers to the average deviation of scores from a person’s mean score (i.e., standard deviation). We estimated relative standard deviations to account for mean differences. Higher scores represent larger average deviations in desire

Women showed significantly greater net variability in desire across 13 years than did men, *β* = 0.18, SE = 0.05, *p* < 0.001, 95% CI = [0.08, 0.28]. See Fig. 2 for the distributions of net variability as a function of sex. However, we found no sex difference in desire instability, *β* = 0.08, SE = 0.05, *p* = 0.111, 95% CI = [ − 0.02, 0.18].

#### Sensitivity Analyses

We found no interactions between number of children and sex on net desire variability (*p* = 0.485) or instability (*p* = 0.235). We also found no significant interactions between sex and a dichotomous variable for whether participants had children or not (*p*s > 0.701). Next, we tested whether our analyses replicated when assessing desire using the two items included across all three waves, and each item separately. Results were consistent with our main analyses for measures of net variability. However, we found significant sex differences in desire instability when desire was measured using single items, *p*s < 0.013. Finally, we tested whether our results replicated when using roughly equal sample sizes of women and men. Results were consistent with our main analyses.

### Discussion

Study 1 largely supports the theory that women’s desire is more variable relative to men’s, at least in the long term. Over a period of many years, women exhibit significantly greater changes in desire compared to men when assessing net variability. We found no effect of gender on desire instability; however, when desire was modeled using individual items, women’s desire was less stable relative to men’s. Results are consistent with previous studies of desire change among newlyweds (McNulty et al., 2019), and couples during the transition to parenthood (Rosen et al., 2021). We note, however, that the size of the sex differences in desire variability was small and the large sample size made it possible to detect even small effects. The distributions of net desire variability for each sex mostly overlap (see Fig. 2), though previous theorizing suggests a marked difference. Thus, Study 1 provides some preliminary support for a small sex difference in the extent to which desire varies over the long term, although this effect may not generalize across all measures of variability.

As an ancillary analysis, we assessed whether sex differences in desire variability may be influenced by childbirth, which has a larger effect on women’s desire relative to men’s desire (Rosen et al., 2021). The effect of sex on desire variability was not significantly moderated by whether participants had children, or the number of children they had.

Since Study 1 sampled a very large proportion of the population over many years, there are some practical limitations to consider. While the sample size for Study 1 was substantial, it had low resolution in the measurement of variability, with 3 time points per person. Estimates of variability were, therefore, constrained to a small number of snapshots of a person’s life. Future studies including additional waves of data collection are needed to assess the extent to which desire exhibits within-person variability over a number of years, the size of the sex difference, and the mechanisms underlying this difference.

It is also important to consider how constructs were measured. Sexual desire was measured using items from the validated Sexual Desire Inventory (Spector et al., 1996). These items tapped both desire for an attractive person and desire for a partner, and results were largely consistent across items and item combinations. The SDI has demonstrated invariance across genders in Columbian (Vallejo-Medina et al., 2020) and Italian samples (Callea & Rossi, 2021), but the English version of the scale has not been tested for gender invariance. Future work is needed to assess whether the English SDI is invariant across genders to rule out the possibility that gender differences are confounded with differences in response styles. It is promising to note that the reliability scores for women and men were very similar, suggesting that the items were similarly related for women and men. Further, Study 1 assessed participants’ assigned sex, rather than gender. Thus, our findings specifically address assumed sex differences in desire variability. Additional data assessing gender and sex would allow for a more comprehensive picture of how gender and sex may influence sexual desire variability.

The study of within-person change in desire over a period of many years is practically challenging, and such longitudinal data tends to be rare. Our models of desire variability over the lifespan are still developing. However, multiple assessments of desire over a short period of time (i.e., intensive longitudinal data) are more accessible and can contribute to a clearer picture of sex differences in short-term desire variability.

## Study 2

Sexual desire can change from year to year, day to day, and moment to moment. At present, there is evidence for a sex difference in medium- and long-term desire variability; however, there is limited evidence that women’s desire is more variable than men’s desire over the short term. In Study 2, we address this gap in the literature by directly comparing women’s and men’s desire variability over the short term. We expand on our analyses of desire variability by including a third measure of variability—inertia. Due to the larger number of time points per individual, we can model the extent to which desire is associated with preceding desire ratings over time*.* We used experience sampling methodology to measure sexual desire multiple times per day over one week among couples. We also measured respondents’ momentary happiness, stress, and tiredness, and momentary relationship states of anger toward their partner, closeness to their partner, dependence on their partner, and relationship satisfaction. We could therefore address RQs 2 and 3 by assessing whether women’s desire was more closely tied to general feelings and feelings about the relationship relative to men’s desire.

### Method

#### Participants

Participants were 115 heterosexual couples (*N* = 230; *M*_age_ = 24.02, SD_age_ = 4.80), ranging in age from 18 to 40 years. Participants were recruited via newspaper advertisements asking heterosexual couples to take part in a study, as part of a broader US-based project examining relationship goals. Participants interested in the study were directed to an initial screening questionnaire. Participants were eligible to participate if they were in an exclusive heterosexual relationship, had been together for at least three months, were aged 18 or older, were fluent in English, and owned a smartphone. The analyses reported in this study are based on data that were collected in the Relationships and Goals Experience Sampling (RELGOES) Study, a large experience sampling project on relationship processes and self-regulation (for details, see Hofmann et al., 2015). The research questions, analyses, and conclusions reached in this paper do not overlap with prior reports.

The average relationship length was 2.61 years (SD = 2.83). In Study 2, 53.9% of participants were White, 16.1% African-American, 16.1% Hispanic/Latino, 12.2% Asian, 0.9% American Indian, and 0.9% other backgrounds.

Six participants dropped out due to technical problems, leaving a final sample of 224 participants (49.1% men). Collectively, participants responded to 6,615 daily surveys (70.3% response rate) and the average response rate was similar for men (69.90%) and women (70.72%).

#### Procedure

Participation involved completing a pre-survey, six surveys each day, nightly surveys, and a post-study survey. Participants were paid $30 if they completed the pre-survey and at least 39 of the possible daily and nightly surveys. In this study, we focus on the relevant measures included in the pre-survey and data from the six daily surveys to assess moment-to-moment changes in desire. Desire was not measured in the nightly surveys. Participants were sent six daily surveys to their smartphones each day for seven days, which were randomly distributed between 9am and 8 pm. Daily surveys within each dyad were yoked such that partners received signals simultaneously. Each survey took 1–2 min to complete.

#### Measures

##### Pre-Survey

Participants completed a demographic survey and other psychological measures. Participants were asked “what is your sex?” with options, “male” and “female.” All participants identified as female or male. Again, we refer to participants as “women” and “men,” but note that the sample may have included participants whose gender identity may have differed from their sex.

##### Experience Sampling

Partner-specific desire was measured using a single item: “How much sexual desire do you feel for your partner right now?” Four items measured the extent to which participants felt happy, mentally exhausted, physically exhausted, and stressed. Because mental and physical exhaustion are overlapping constructs, both conceptually and statistically (*r* = 0.59, *p* < 0.001), they were combined to form a single “tiredness” scale. We use the umbrella term “affective states” for concision; however, we note that stress and tiredness are not strictly emotions (Lazarus, 1990).

Four items measured relationship-oriented states, including the extent to which participants felt satisfied with their relationship, close to their partner, angry with their partner, and reliant on their partner. Response options for experience sampling questions ranged from 0 to 6.

#### Analytic Strategy

We assessed sex differences in net desire variability and instability using the same procedure as Study 1. Participants were excluded from analyses if they only provided one measurement of sexual desire (*n* = 3). We also excluded participants from analyses of net variability and instability if their mean desire was equal to the lower bound of the scale (*n* = 0) or the upper bound of the scale (*n* = 2). This left 219 participants for the analysis of net variability and instability.

We also assessed a third measure of variability—desire inertia. Unlike net variability and instability, inertia captures the extent to which desire may be resistant to change or is persistent over time. We calculated inertia using an autoregressive (AR1) multilevel model. We calculated a person-mean centered lagged desire variable, to capture within-person change in desire. The lagged desire variable was regressed onto desire measured at each time point, including a random intercept and slope for participant. The autoregressive (AR) slope represents the extent to which desire at one time point is predictive of desire at the previous time point (i.e., inertia). To estimate sex differences in inertia, we modeled the interaction between sex and the autoregressive effect of desire across time points.

To assess the extent to which momentary desire varies as a function of other momentary states, we calculated actor effects for women and men. Actor effects assess the extent to which predictor variables (e.g., anger) are associated with an outcome (e.g., desire). We tested whether women’s and men’s actor effects significantly differed from each other to test whether affective/relationship states had a larger effect on women’s desire relative to men. These analyses addressed the question: Is women’s desire more sensitive to affective and relationship states relative to men’s desire?

We also calculated partner effects—the extent to which desire varies as a function of a partner’s affective/relationship state. Again, we tested whether partner effects between women and men were significantly different. These analyses addressed the question: Is women’s desire more sensitive to their partner’s affective states relative to men’s desire?

We used multilevel models for repeated intensive longitudinal dyadic data with distinguishable dyads, following Bolger and Laurenceau (2013), to account for the nesting of time points (*N* = 6,656) within persons (*N* = 224) within dyads (*N* = 112).

### Results

Mean sexual desire across the 6,656 data points was at the midpoint; *M* = 2.99, SD = 2.00. Men’s desire (*M* = 3.14, SD = 2.02) was slightly higher than women’s desire (*M* = 2.84, SD = 1.96), but it was not a statistically significant difference, *β* = -0.13, SE = 0.08, *p* = 0.104, 95% CI = [-0.29, 0.03].

#### Desire Variability

**Fig. 3:**
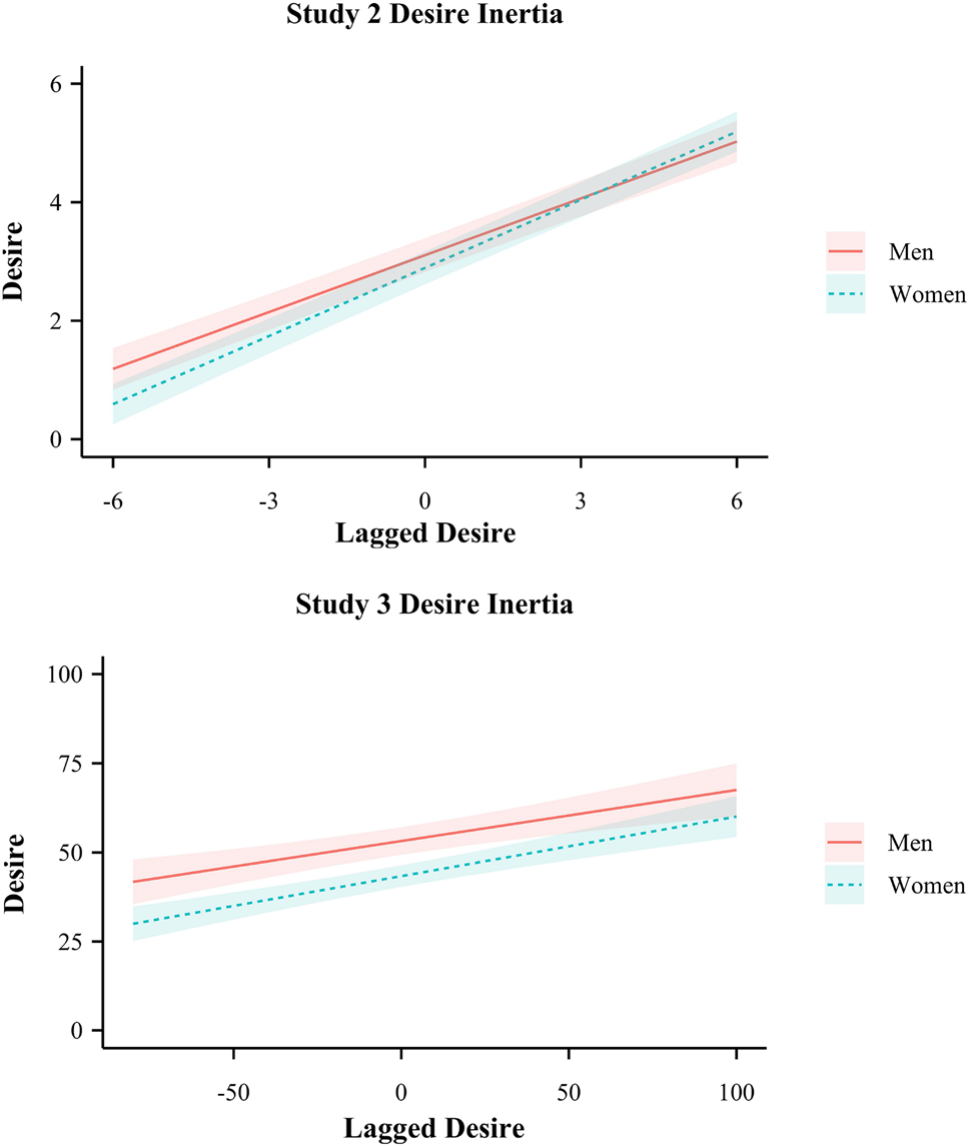
Desire inertia for women and men in Studies 2 and 3. *Note*: desire inertia refers to the extent to which a person’s desire at one time point is associated with their desire at the previous time point. A steeper slope indicates that desire is more similar over time. Lagged desire was person-mean centered

As in Study 1, we estimated the effect of sex on net variability and instability. In contrast to Study 1, and pertinent to our first research question, we found no effect of sex on net desire variability (*β* = − 0.08, SE = 0.14, *p* = 0.552, 95% CI = [ − 0.35, 0.19]). We found no moderating effects of age (*p* = 0.227) or relationship length (*p* = 0.480). There were no significant sex differences in variability across the other states (*p*s > 0.059). Variability in desire tended to be at the higher end (SD = 2.00) relative to other affective states (SDs ranged from 1.20 to 1.98), suggesting that the null effect of sex on variability was not a result of floor effects. We also found no effect of sex on desire instability, *β* = − 0.20, SE = 0.13, *p* = 0.148, 95% CI = [ − 0.46, 0.07]. The interaction between sex and desire inertia was also non-significant, *β* = 0.04, SE = 0.02, *p* = 0.101, 95% CI = [ − 0.01, 0.08] (see Fig. 3).

#### Actor and Partner Effects of Affective and Relationship States on Desire

To assess the effect of affective and relationship states and a partner’s affective and relationship states on desire, we conducted actor–partner interdependence models. Actor and partner effects are reported in Tables 1 and 2, respectively. In terms of actor effects, feeling angry and stressed were associated with lower desire for both men and women, while feeling happy, close to one’s partner, satisfied with one’s relationship, and dependent on one’s partner were associated with higher desire for men and women. There was a negative association between tiredness and desire for women but not men. In terms of significant sex differences between these associations, the associations between desire and anger toward a partner and desire and tiredness were larger for women. There were no other significant sex moderation effects.

**Table 1:** Actor effects of affective and relationship states on sexual desire and differences in effect size for women and men: Study 2. Unstandardized coefficients are reported with standard errors

	Women				Men				Gender difference		
	*b*	SE	*p*	95% Cis [lower, upper]	*b*	SE	*p*	95% Cis [lower, upper]	Mean diff.	SE	*p*
Anger	− .41	.04	< .001	[ − .492, − .320]	− .29	.03	< .001	[ − .353, − .225]	− .12	.06	.047
Close to partner	.55	.03	< .001	[.485, .622]	.51	.04	< .001	[.439, .578]	.05	.05	.335
Dependent on partner	.33	.03	< .001	[.269, .396]	.32	.03	< .001	[.262, .373]	.01	.05	.754
Happy	.36	.03	< .001	[.297, .421]	.34	.03	< .001	[.282, .401]	.02	.04	.685
Relationship satisfaction	.54	.04	< .001	[.458, .619]	.56	.04	< .001	[.475, .638]	− .02	.06	.752
Stress	− .18	.03	< .001	[ − .246, − .112]	− .18	.03	< .001	[ − .235, − .120]	< − .01	.05	.968
Tired	− .17	.03	< .001	[ − .233, − .099]	− .01	.03	.746	[ − .094, .008]	− .16	.04	< .001

**Table 2:** Partner effects of affective and relationship states on sexual desire and differences in effect sizes for women and men: Study 2. Unstandardized coefficients are reported with standard errors

	Women				Men				Gender difference		
	*b*	SE	*p*	95% Cis [lower, upper]	*b*	SE	*p*	95% Cis [lower, upper]	Mean diff.	SE	*p*
Anger	− .10	.04	.008	[ − .169, − .026]	− .10	.03	.001	[ − .160, − .041]	< .01	.05	.933
Close to partner	.13	.03	< .001	[.072, .192]	.09	.03	.002	[.034, .146]	.04	.04	.348
Dependent on partner	.08	.02	< .001	[.269, .396]	.09	.02	< .001	[.042, .136]	− .01	.03	.778
Happy	.14	.02	< .001	[.089, .185]	.10	.02	< .001	[.047, .144]	.04	.04	.246
Relationship satisfaction	.18	.04	< .001	[.109, .256]	.13	.03	< .001	[.067, .183]	.06	.05	.238
Stress	− .09	.02	< .001	[ − .130, − .047]	− .07	.03	.010	[ − .122, − .047]	− .02	.04	.598
Tired	− .04	.02	.065	[ − .091, .003]	− .04	.03	.101	[ − .094, .008]	< − .01	.04	.972

In terms of partner effects, effects for women and men were consistent. For women and men, partner stress and anger were negatively associated with desire. Similarly, partner happiness, closeness, perceived dependence, and relationship satisfaction were positively associated with desire. There were no partner effects of tiredness for women or men. We found no significant differences in the size of these partner effects for women and men.

### Discussion

In Study 2, we found no effect of sex on desire variability in the short term. With regard to RQs 2 and 3, we find some sex differences in the degree to which desire for one’s partner is influenced by other affective and relationship-oriented states. Specifically, in terms of affective states, feeling tired is associated with significantly lower desire for women, but not for men. Further, the size of the negative association between tiredness and desire was significantly larger for women relative to men. In terms of relational states, feeling angry with one’s partner has a stronger negative association with women’s desire relative to men’s desire.

Study 2 provided evidence that sex differences in desire variability may depend on the time scale—from moment to moment, women and men appear to vary in their desire to a similar degree. Our findings were mixed regarding a sex difference in desire sensitivity to other affective and relationship states, suggesting that women’s desire may be more sensitive to some, but not all, situational factors.

Study 2 assessed desire for a partner among people in relationships, and with a relatively young sample. It may be that desire for a partner oscillates to a similar degree for women and men in relationships, where there are similar stimuli influencing desire. However, general desire, not necessarily desire for a partner, may be more context-sensitive for some people than others.

## Study 3

In Study 3, we tested for sex differences in desire over the short term, as we did in Study 2. However, in Study 3, we assessed general desire, not partner-specific desire, among a sample of people in relationships and not in relationships. Study 3 therefore captured variability in the extent to which desire in general can shift from moment to moment. Study 3 also tested whether relationship status moderates the association between sex and desire variability. Further, Study 3 sampled a broader age range, allowing for a better test of the moderating effect of relationship length on the association between sex and desire variability. We used experience sampling methodology to measure sexual desire, feelings of attractiveness, stress, tiredness, closeness to their partner, and loneliness.

### Method

#### Participants

We gathered 4,667 observations over the course of a week. These observations were drawn from ninety-five men and 160 women (*M*_age_ = 31.76, SD_age_ = 10.45), ranging in age from 18 to 64 years. In total, 472 people clicked the link to our online pre-survey and entered a personal identifier, and 361 people downloaded the mobile app and entered a personal identifier. Of those, 258 people entered matching personal identifiers in the pre-survey and app survey. Three people were excluded because they identified their sex as “other” (see General Discussion for engagement with this issue). The majority of participants were in a relationship (73%) with an average relationship length of 6.41 years (SD = 7.31). The sample was predominantly White/Caucasian (88%), with 8% Asian, 1% Hispanic, and 3% “Other.”

#### Procedure

Participants were invited to participate via a number of online channels, including Australian news websites and university advertising. After completing the pre-survey, we asked participants to download the study app to their smartphone. The app delivered 4 randomly timed surveys each day for 7 days. Study 3 sent fewer signals per day compared to Study 2, in part to reduce participants’ burden and minimize attrition, as participants were not financially compensated for their time. Participants completed a total of 4,667 daily surveys. On average, participants completed 18.3 daily surveys (SD = 6.77) of a possible 24–28 (participants who signed up after the last notification on any given day received notifications for the remaining 6 days). The average number of responses was similar for women and men (*M*_women_ = 18.16; *M*_men_ = 18.55).

Survey prompts were scheduled from 8am to 9 pm on weekdays and 10am to 8 pm on weekends. Participants were instructed to “complete the surveys as soon as you receive them, or as soon as possible.” Surveys had to be completed within 2 h of receipt of the prompt, and survey prompts were separated by at least 2 h. The incentive to complete the study was non-monetary; upon completion of the study, participants had the option to view a personalized summary of results.

#### Measures

##### Pre-Survey

Prior to participating in the experience sampling study, participants reported their demographics and responded to attitudinal measures. Sex was assessed using the question, “what is your sex?” (“female,” “male,” “other”). Again, we refer to “women” and “men,” but note that we measured sex, and so the sample may include participants who are not cisgender.

##### Experience Sampling

Momentary sexual desire was measured using two items: “How much do you feel like having sex at this moment?” and “How would you rate your current level of sexual desire or interest?” The second item was adapted from the Female Sexual Function Index (Rosen et al., 2000). These items were highly correlated, so were combined to form a scale (*r* = 0.88, *p* < 0.001). When analyzed separately for men and women, the correlation between the two items was very similar (*r*_*men*_ = 0.89, *p* < 0.001; *r*_*women*_ = 0.86, *p* < 0.001). Three items measured the extent to which participants felt tired/alert (reversed), stressed, and attractive. Two items measured relationship-oriented states—the extent to which participants felt emotionally close to their partner (participants not in a relationship were asked to skip this question) and lonely. Scales ranged from 0 to 100. Items were presented in random order.

##### Analytic Strategy

We assessed sex differences in net variability, instability, and inertia using the same procedure as in Study 2. We excluded one participant with a mean desire of zero and one participant who had only responded at one time point. This left 253 participants for the analyses of net variability and instability.

As in Study 2, we conducted multilevel analyses in R to test associations between predictor variables and sexual desire. Relevant to RQs 2 and 3, we assessed the moderating effect of sex. Responses at each time point (Level 1; *N* = 4,667) were nested within-person (Level 2; *N* = 255). The intra-class correlation (ρ_ICC_ = 0.42) indicated that within-person variation was substantial, accounting for 58% of the total variance in sexual desire.

In order to test our second and third research questions regarding whether psychological states have a stronger effect on women’s desire, we conducted a series of univariate multilevel models (with random intercepts and slopes for participant) predicting current desire as a function of affective states (feeling attractive, stressed, and tired) and relationship-oriented states (partner closeness and loneliness), moderated by sex. Participants not in a relationship were excluded from analyses on partner closeness but were included in all other analyses. Results remained unchanged after excluding participants not in relationships. Continuous predictor variables were standardized and person-mean centered.

### Results

Mean sexual desire across the 4,667 data points was close to the midpoint; *M* = 46.13, SD = 27.56. Men’s desire (*M* = 53.12, SD = 26.65) was significantly higher than women’s desire (*M* = 41.89, SD = 27.24), *b* = -0.36, SE = 0.08, *p* < 0.001.

#### Desire Variability

There was no significant sex difference in net variability over 7-days (*β* = 0.17, SE = 0.13, *p* = 0.192, 95% CI = [ − 0.09, 0.43]). The interaction between sex and age on net variability was non-significant (*β* = − 0.18, SE = 0.13, *p* = 0.150, 95% CI = [ − 0.44, 0.07]). We also tested the interaction between sex and relationship length among participants in relationships (*n* = 182). The interaction between sex and relationship length, controlling for age, was again non-significant (*p* = 0.135). Finally, we tested the interaction between relationship status and sex, which was also non-significant (*p* = 0.909). Interestingly, sexual desire was one of the few momentary states for which we did not see sex differences in variability: women were more variable than men in their feelings of attractiveness, partner closeness, and stress (ps < 0.040). Desire variability was relatively large (SD = 27.60) compared to other momentary general affective states (SDs: 20.69–25.05), suggesting that the null effect of sex on variability was not a result of floor effects. We also found no effect of sex on desire instability, *β* = 0.19, SE = 0.13, *p* = 0.134, 95% CI = [ − 0.06, 0.45]. The interaction between sex and desire inertia was also non-significant, *β* = 0.02, SE = 0.03, *p* = 0.555, 95% CI = [ − 0.04, 0.08] (see Fig. 3). See Fig. 1 for examples of participants with high and low desire net variability, instability, and inertia.

#### Desire and Affect

We found significant relationships between desire and all affective and relationship-oriented states (see Table 3). Importantly, sex did not significantly moderate any of these relationships, *p*s > 0.364. There was a slightly stronger association between feeling attractive and feeling sexual desire for men relative to women; however, the effect was not significant, *p* = 0.056.

**Table 3:** Relationships between desire, general affective states, and relationship-oriented states, and interactions with gender: Study 3

	*β*	SE	*p*	95% Cis [lower, upper]
*Univariate multilevel models*				
Feeling attractive	0.25	0.02	< .001	[0.214, 0.277]
Tired	− 0.14	0.02	< .001	[0.113, 0.174]
Stressed	− 0.19	0.02	< .001	[ − 0.216, − 0.157]
Lonely	− 0.10	0.02	< .001	[ − 0.127, − 0.065]
Close to partner	0.21	0.02	< .001	[0.172, 0.246]
*Univariate multilevel models with gender interaction*				
Feeling attractive × gender	− 0.07	0.03	.056	[ − 0.133, 0.001]
Tired × gender	− 0.01	0.03	.695	[ − 0.051, 0.076]
Stressed × gender	− 0.02	0.03	.581	[ − 0.081, 0.045]
Lonely × gender	− 0.02	0.03	.594	[ − 0.082, 0.047]
Close to partner × gender	− 0.04	0.04	.364	[ − 0.118, 0.043]

Effects are reported controlling for the random effect of person

### Discussion

Study 3 found no significant sex differences in intra-individual variability of desire measured over a week. Relevant to our first research question, desire fluctuated throughout the week, but did so equally for men and women. This effect was not moderated by age or relationship length. Second, the effects of affective states on desire were similar for men and women. The finding that sex did not moderate the effect of tiredness on desire differed from Study 2, where tiredness had a stronger negative effect on women’s desire relative to men’s. Third, relationship-oriented states such as feeling close to one’s partner and feeling lonely were strong predictors of desire, but again, sex did not moderate these effects. In Study 3, we find striking consistency in men’s and women’s fluctuations in sexual desire over seven days.

A key difference between Studies 2 and 3 is the measure of desire. It may be that, in the short term, women’s desire for a partner is more sensitive to some negative states relative to men’s desire, whereas associations between affective and relationship states and *general* desire are more similar for women and men.

## General Discussion

Social psychological and lay theories of desire suggest that men’s desire is stable (and high), whereas women’s desire ebbs and flows depending on their social context (Baumeister, 2000; Regan & Berscheid, 1995). The present study sought to test this assumption, specifically addressing the following three questions: (1) Do women show more variability in sexual desire compared to men? (2) Compared to men, is women’s sexual desire more strongly related to their general affective states? And (3) Is women’s sexual desire more strongly tied to their relationship-oriented states compared to men’s desire?

In order to assess intra-individual changes in desire, we conducted three longitudinal studies assessing desire over 13 years (Study 1), and from moment to moment over 7 days (Studies 2 and 3). These studies collectively sampled women and men at 16,885 time points using diverse sampling methods, including community samples in Australia and the US and a population-based sample in Finland. We assessed general desire in Studies 1 and 3 and partner-specific desire in Study 2.

With regard to our first research question, when desire was measured over the longer term, women’s desire varied to a greater extent than men’s desire. In Study 1, desire was assessed three times over 13 years, with women showing significantly greater variability than men, consistent with previous research assessing changes in desire among newlyweds over four years (McNulty et al., 2019), and studies of desire during the transition to parenthood (Rosen et al., 2021). Thus, the theory that women’s desire is more variable than men’s desire is supported by longitudinal studies examining desire over many years. However, the effect was small, as can be seen in Fig. 2, and Study 1 was the most well-powered study to detect a small effect. As is often the case, the overlap in women’s and men’s distributions far exceeds the differences.

Study 1 raises the question of why desire may be more variable among women compared to men over the long term. One possibility, supported by Rosen et al. (2020), is that the transition to parenthood has a larger impact on women’s desire relative to men. We did not, however, find a significant interaction between sex and having children on desire in Study 1 (cf. McNulty et al., 2019; Rosen et al., 2021). A second possibility is that women’s desire may be more likely to decline with age as a function of feeling less attractive, due to the intersecting experiences of gendered beauty standards and ageism (Buote, 2010; van Anders et al., 2021). Relatedly, it may be that women’s desire is more likely to change over time as a function of relationship inequities if they are partnered with a man (Harris et al., 2022; van Anders et al., 2021). Additional waves of data and/or additional longitudinal studies are needed to further test the extent to which women’s desire varies more than men’s over the lifespan, and why.

When desire was measured over the short term, our results diverged from previous theorizing and quantitative results—we found no evidence that women’s desire was more variable than men’s desire in the short term. On average, both men and women show relatively large fluctuations in desire over seven days. Academic and lay assumptions about women’s desire being variable appear to be accurate. However, the assumption that men have stable desire was not supported by the data, at least in the short term. Men’s desire was as variable as women’s desire, and it was more variable than other states, such as stress and tiredness. Our findings suggest that female erotic plasticity theory, therefore, may not extend to desire in the short term.

To assess the second and third research questions, we tested whether sex moderates the associations between desire and affective and relationship-oriented states “in the moment.” In terms of affective states and desire, women and men showed similar patterns. The associations between desire and stress, attractiveness, happiness, and loneliness were significant and not moderated by sex. These findings counter assumptions that women’s desire is more sensitive to contextual factors compared to men. In particular, feeling attractive or satisfied with one’s body is often tied to women’s sexuality. Our findings suggest that researchers and the lay public may underestimate the importance of feeling attractive for men’s desire, consistent with qualitative research from Murray and Brotto (2021) showing that men in heterosexual relationships “desire to feel desired.” The effect of tiredness on desire was stronger for women in Study 2, but not in Study 3. Thus, while women’s desire was sensitive to their immediate affective states, men’s desire was equally so, perhaps with the exception of feeling tired.

In terms of the associations between relationship states and desire, there were some differences between men and women. Across Studies 2 and 3, we found no moderating effect of sex on the associations between desire and feelings about their relationship, with one exception. In Study 2, women’s anger towards their partner were more strongly (negatively) associated with desire than men’s. Thus, there may be some nuanced differences in the extent to which affective and relationship states are associated with desire. Overall, however, the patterns of association were strikingly similar for women and men, with only two of nineteen relationships moderated by sex.

### Implications

Our findings provide an opportunity to build upon our current models of sex and desire over time. While the theorizing around women’s variability in desire is supported in the longer-term, it does not apply to moment-to-moment changes in desire. These findings support a distinction between short-term changes, or “state” desire, and medium- to long-term changes, or “trait” desire. Factors affecting desire “in the moment” may diverge from those affecting desire in the long term, consistent with work on gender and sex differences in absolute levels of desire (Dawson & Chivers, 2014).

Factors affecting momentary desire may also diverge from those affecting other fluid dimensions of sexuality. Women appear to show greater variability in their sexual attitudes, behavior, and attraction (e.g., Diamond et al., 2017) compared with men, but not desire, at least in the short term. One possible explanation is that desire is experienced similarly to other mood states, such as hunger or tiredness. As such, desire may be more likely to vary along with other momentary states, rather than individual differences in sex or gender. Other dimensions of sexuality, such as attitudes, behavior, and attraction, may be more sensitive to gendered pressures and expectations. Additional theorizing and research are needed to assess the relative influence of gendered expectations across different dimensions of sexuality.

Our findings regarding short-term desire variability have notable practical implications for women’s and men’s sexual self-concepts and sexual relationships. An assumption that men have stable desire and women have fluctuating desire may lead to inaccurate impressions of the world—that is, we may perceive women to be “hot and cold” and simultaneously underperceive men’s variability in desire. We may also discourage men from acknowledging fluctuations in desire if they are felt to be “not manly,” and men may subsequently engage in sexual activity despite experiencing a period of low sexual interest. Finally, for women partnered with men, sexual rejection may be more painful if women assume men have consistently high desire. That is, if a woman is under the assumption that her man partner has a consistently high sex drive, his disinterest in sex is likely to be attributed to external factors (such as her desirability) rather than internal factors (such as his naturally fluctuating sex drive). This may partially explain why women tend to have more negative responses to sexual rejection compared to men (de Graafe & Sandfort, 2004) and speaks to the importance of communication when engaging in sexual rejection (Impett et al., 2020). As such, acknowledging that desire changes in both men and women may diminish negative feelings in response to a partner’s sexual disinterest.

### Limitations and Future Directions

The study of within-person changes in sexuality is still in its infancy. Research on desire discrepancy has shed light on the variable nature of desire—desire fluctuates, and these fluctuations are likely going to be different between partners, such that one partner may peak while another partner may drop (Mark, 2012, 2014; Ridley et al., 2006). Daily diary studies have uncovered practices and strategies that can “keep the spark alive,” buffering against drops in desire over time (Muise et al., 2013b). These previous studies, and findings from Studies 2 and 3, support a “state” conceptualization of desire, whereby desire can fluctuate throughout the day and in response to external events. Further, women’s and men’s desire appear to be equally “state-like,” such that variability in desire is similar for women and men in the short term.

We note, however, that our findings are specific to our conceptualization of desire as a state. We assessed desire using one to three items that were designed to assess a brief snapshot of a person’s current level of desire. The items tended to be highly correlated (Studies 1 and 3), and single-item measures demonstrate appropriate predictive validity in experience sampling studies (Song et al., 2022). Further, our findings are largely consistent with previous work conceptualizing desire in response to sexual stimuli, whereby patterns of desire change are similar for women and men (Dawson et al., 2013). As such, this study directly addresses sex differences in state desire variability.

We did not explicitly assess trait desire, so our findings cannot speak to the extent to which women’s and men’s trait levels of desire change over time. Previous work has found that gender and sex differences in average desire may be more likely to emerge when desire is conceptualized as a trait rather than a state (Dawson & Chivers, 2014). Thus, it may be that when operationalized as a trait, desire may be more stable and trait-like for men than women. However, there is an open question as to whether it is appropriate to conceptualize desire as a trait (Dawson & Chivers, 2014; Mark & Lasslo, 2018).

The measurement of state desire may be appropriate given the extent to which it fluctuates, however, our measures may be constrained in other ways. It is possible that participants in our studies were responding according to the demand characteristics of the studies. In Study 3, we controlled for social desirability and found that the results remained unchanged. An additional possibility is that desire levels were inflated by virtue of completing the daily surveys—that is, being asked to introspect on one’s desire may cause an increase in desire. While we think this is a possibility, we do not believe this would affect our conclusions, as we did not find ceiling effects of desire, and we were interested in sex differences in variability in desire rather than baseline levels of desire.

Finally, our findings speak more directly to theories relating to sex differences in desire variability. Additional data are needed to assess whether our results would hold when assessing participants’ gender. Further, studies of gender and sex differences tend to focus on women/females and men/males and tend to only sample, heterosexual participants. Future research is needed to explore gender and sex differences beyond the gender binary, and why differences may exist. For non-binary and/or allo-binary participants, it may be that desire is sensitive to contexts in which gender identity is affirmed or denied, which might in turn influence relevant affective states, such as happiness, attractiveness, and partner closeness. And, of course, this may be similar for men and women who do identify within the binary. It may be that heterosexual women’s desire varies over the life span, as a function of heteronormative pressures, whereas women not partnered with men may experience desire differently. Future research on experiences of desire with gender and sexually diverse samples is needed to help answer these questions and contribute to a growing field of feminist and queer research on desire (e.g., Chadwick et al., 2017; Holmberg & Blair, 2009; Mark et al., 2018).

### Conclusions

Men’s sexual experience is more likely to be discussed in terms of natural, biological urges that lead to unwaveringly high sex drives. Women’s sexuality, on the other hand, is viewed as relational and socially responsive (Regan & Berscheid, 1995). While neither of these descriptions is necessarily good or bad, they appear to exaggerate sex differences in women’s and men’s sexuality. In the present studies, we find that patterns of desire are remarkably similar for men and women when measured over the short term, although there is some evidence that women may show greater variability in desire over the longer term. Men, just like women, fluctuate in the degree to which they desire sex, and are equally impacted by general affective states (such as how stressed they feel) and a number of relationship-oriented states (such as how close they feel to their partner). In short, men’s desire may be more malleable and sensitive to social factors than previously thought, with implications for theory as well as interpersonal dynamics in relationships.

## Data Availability

Analysis code and output for Studies 1–3 are available via OSF (https://osf.io/vsxwb/?view_only=fd32a4fe21d54631a30d7a93474154c9).

## Footnotes

1 The sample of participants in non-heterosexual relationships was too small to assess differences in desire variability between partners.
